# Malthusian Trajectory for Heart Failure and Novel Translational Ambulatory Technologies

**DOI:** 10.2174/1573403X18666220524145646

**Published:** 2023-03-22

**Authors:** Biddargardi Niranjan, Maximilian P. de Courten, Pupalan Iyngkaran, Malcolm Battersby

**Affiliations:** 1 Digital Health at College of Medicine and Public Health Flinders University & SAHMRI, Adelaide, Australia;; 2 Mitchell Institute for Education and Health Policy, Victoria University, 300 Queen St, Melbourne, Australia;; 3 Mitchell Institute, Victoria University, Melbourne, Australia and Werribee Mercy Sub School, School of Medicine Sydney, The University of Notre Dame Australia, Werribee, Australia;; 4 College of Medicine and Public Health, South Australian Health and Medical Research Institute, Southern Adelaide Local Health Network, Mental Health Division, Flinders Medical Centre, Flinders University, Adelaide, Australia

**Keywords:** Ambulatory care, congestive heart failure, health, technology, translation, malthusian concept

## Abstract

**Introduction:**

It has been estimated that congestive heart failure (CHF) will reach epidemic proportions and contribute to large unsustainable impacts on health budgets for any cardiovascular condition. Against other major trends in cardiovascular outcomes, readmission and disease burden continue to rise as the demographics shift.

**Methods:**

The rise in heart failure with preserved ejection fraction (HFpEF) among elderly women will present new challenges. Gold standard care delivers sustainable and cost-effective health improvements using organised care programs. When coordinated with large hospitals, this can be replicated universally.

**Results:**

A gradient of outcomes and ambulatory care needs to be shifted from established institutions and shared with clients and community health services, being a sizeable proportion of CHF care.

**Conclusion:**

In this review, we explore health technologies as an emerging opportunity to address gaps in CHF management.

## 1 INTRODUCTION

Health technologies are the cornerstone of therapy in congestive heart failure (CHF) and medical practice. Defining gaps and novel technologies is difficult, as there are many layers to clinical care and health systems. For example, there are established technologies translated for clinical use, with regular updates and versions; there are established technologies with implementation gaps relating to needing models of care rather than the technology itself, and there are entirely novel technologies where clinical utility remains unevaluated. Technologies and their user interface fundamentally differ when the purpose is related to health or medical indications. This difference must be an integral consideration in health technology developments. It is an issue of health policy at a population level. The scope includes safeguarding privacy, sharing information, funding models, and selecting outcome measures for efficacy trials. The latter emphasizes cost-effectiveness, aiding the clinical translation of findings [1-3].

Over decades, the traditional acute CHF treatment model of prolonged bedrest has evolved into short hospital stays (or frequent early outpatient reviews), followed by a chronic ambulatory phase. Treatment involves a combination of pharmaceuticals, devices, and allied health support. This model works well. However, there are aspects of this model that are costly and labour intensive and cause stress on health budgets and health resourcing. The newer phase of novel health technologies has emerged to address these issues within the established CHF or chronic care model (CCM). However, medical services and information have different sensitivities than liberal rules with social uses. This means the boundaries of doctor-patient encounters, factoring, and technology have never seen a satisfactory solution to allow the full breadth of technological achievements to prosper [1-4]. Among its greatest strengths is bridging Malthusian concepts of resourcing and population growth. Nolan *et al.* [2] discussed technologies that focus on CHF. In this perspective, we explore the Malthusian concept of growth along with data and communication gaps.

## 2 MATERIALS AND METHODS

### 2.1 Heart Failure Trajectory, Health Technology Gains, and Obstacles

In literature, the invaluable need for technology has been consolidated. At a similar juncture in the late 1700s, Thomas Malthus's writing, “Essay on the Principle of Population,” suggested an exponential growth in population and linear growth in food production [5]. The novelty of technology is the trajectory of its use that can never be predicted, and it is to overcome the related challenges. Thus, while Malthus's question can be solved through novelties in ideas and medicine, the bridges to cross include technology as well as the purpose and ability to use it (*i.e.*, socio-political considerations).

#### 2.1.1 Heart Failure as an Epidemic

The role of novel technologies is evident in CHF epidemiology. In the United States, the prevalence of CHF is >5.7 million, with 670 000 new cases yearly. In Europe and globally, the prevalence is >15 million and 37.7 million, respectively. It is the leading cause of hospitalization in patients >65 years of age, with more than 1 million primary presentations or 1% to 2% of all hospitalizations yearly. Annual medicare expenditure in the United States exceeds US $17 billion. Following a CHF admission, 1 in 4 are readmitted within the first month and a half within 6 months, where 80% of emergency room presentations are admitted. In addition, presentations, readmissions, and costs for CHF are projected to increase by 50% by 2035 [3]. Both systolic (HFrEF) and diastolic (HFpEF) share similar epidemiology, although there are pathophysiological and clinical differences. When factoring HFpEF and its trajectory, the challenges facing health systems are significant [6].

#### 2.1.2 Obstacles and Cost-effectiveness Considerations

**Fig. (1):**
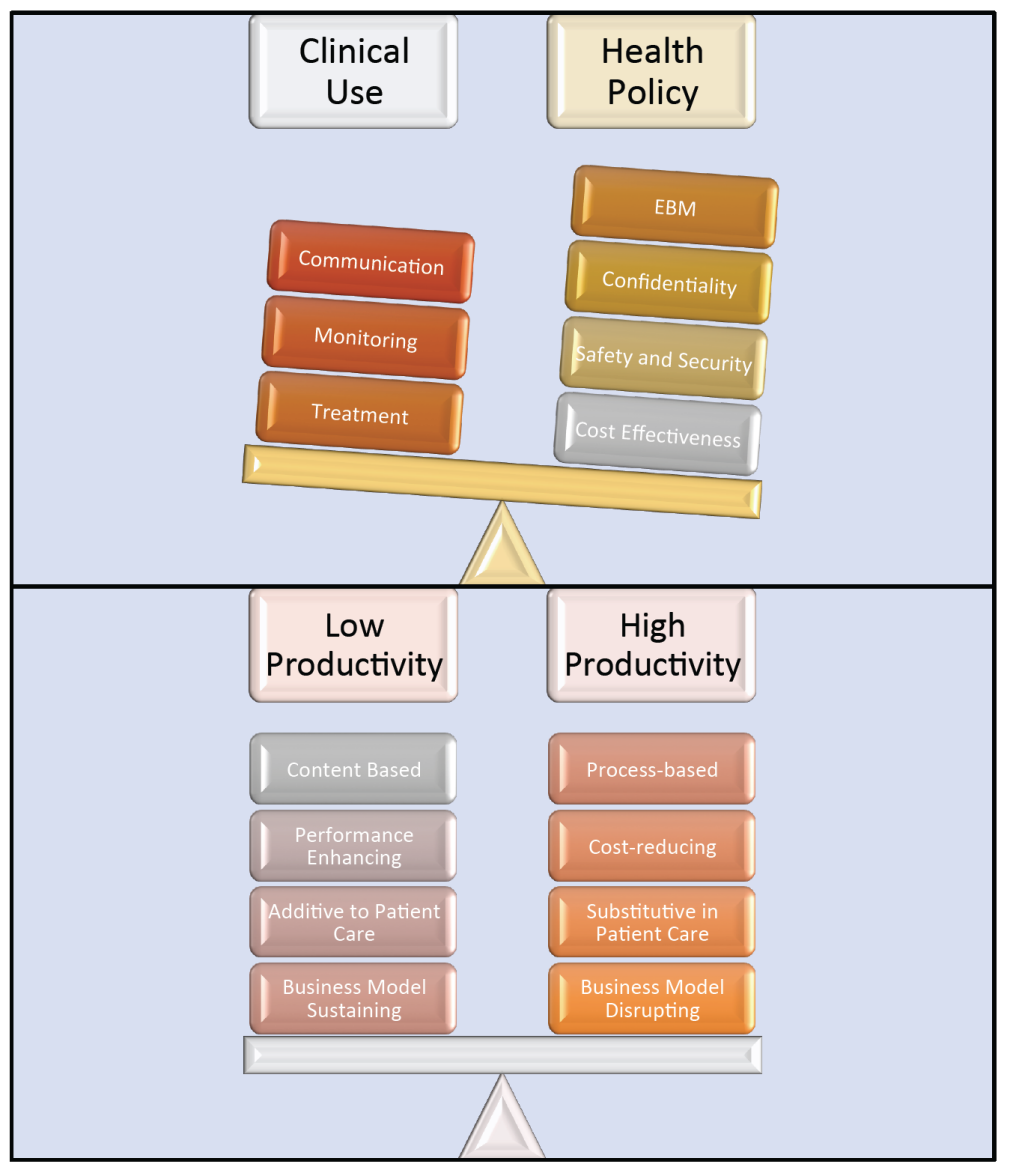
Indication and barriers to heart failure technologies. Lags in health policy create deficits in cost-effectiveness with technologies. Such policy requires societal mandates, and thus implementation varies. The foundations of clinical use are likely to remain the same. However, the choices that are implemented will be determined by confidence in addressing the policy factors. Innovation also has a relationship with growth, expenditure, and outcomes. This balance needs to be weighed [8].

The COVID-19 pandemic revealed an opportunity and highlighted gaps. The opportunity was for technology to fill voids in ambulatory health services. The gaps were noted in the high level of resourcing and the scalability constraints in population-wide patient care. Cahan *et al*. pointed out that the mechanical ventilator invented nearly a century ago faced a bottleneck in human resourcing and health economics [7]. Clearly, this problem can now be matched with more novel thinking. Thus an important barrier to novelty is the novelty in health services thinking. The bench-to-bedside link for evidence-based medicine must similarly be challenged with an *“idea to health policy”* link (Fig. **1**). In this vein, health economics must be given consideration [3, 8, 9].

#### 2.1.3 Technological Mainstay in Routine Heart Failure and Cardiovascular Care

Significant benefits are seen in CHF, most notably in implantable devices and ambulatory diagnostics, such as 24-hour Holter monitors. While not widely implementable, software with health apps and mobile-based services are mainstay technologies that will be integrated with more work (Table **1**). It is vital that these gains be consolidated and innovation continues to ensure that health economics remain healthy.

##### 2.1.3.1 Current Model

a. Communication is based largely on CHF models applied and utilised relevant domains within standardised disease management taxonomies, *e.g.*, Krumholtz’s 8-domain guideline. This includes the delivery tool, such as telephone or email and the personnel, *e.g.*, nurse-led. The evidence for these is now established and in guidelines [10, 11]; valuable outcomes are based on the right selection for the needs of the health system [12].

b. Diagnostics tools in cardiology are advanced and established, and research continues to advance them further. They are worthy of discussion and novel within this context. Remote monitoring is of special importance and is discussed below. Information otherwise is widely available in the literature [13, 14].

c. Therapeutics, particularly implantable devices, have significantly improved prognoses when selected appropriately. Research in personalising, widening indications and hardware and software features will also advance this area [15, 16].

##### 2.1.3.2 Future

a. Considering MHealth, recent evidence supports a lack of benefit in CHF and also poor uptake despite high mobile phone use. Hence, there remain substantial areas to explore [17-20].

b. Immersive health: The concept of reality-simulation training has bridged medicine in training particularly. It has the potential to impact frontline healthcare, therapeutic techniques, and research and development. There are four areas of importance that need to be considered, such as augmented reality, virtual reality, machine learning, and artificial intelligence [21].

## 3 RESULTS AND DISCUSSION

### 3.1 Data and Communication Gaps

Digital health refers to the application of information and communication technologies to address gaps in healthcare and also integrate health, well-being and social awareness in technologies that have become part of everyday living. Over recent years, digital technologies have rapidly advanced and transformed various aspects of everyday life and society. As a result, the availability of information sources and mediums of reach and communication have vastly increased in volume, variety and velocity. From a healthcare perspective, emerging information and communication channels provide significant opportunities to devise digitally enabled approaches to address access barriers encountered by both patients and health professionals in the management of chronic heart failure. Importantly, patient assessment and support paradigms can shift from periodic clinic-based to a naturalistic living environment, real-time and longitudinally assessment. Here, we illustrate some aspects with examples of applications.

### 3.2 Information and Data Sources

#### 3.2.1 Electronic Health Records and Data Linkage

The increasing uptake of electronic health records by different parts of the health system, in conjunction with advances in capabilities to link, organise and present data, provides an unprecedented opportunity to improve care for patients and populations. Providing health professionals with access to a secure and quick summary of patient health information is the primary driver behind the digitisation of health records. However, integrated electronic health records have also become the digital footprint of chronic patients as they interact and move between GPs, hospitals, specialists, allied health, pharmacy, and other care providers. This data opens up new opportunities. By algorithmically auditing patients' appointments, tests and medications against guidelines and treatment plans using automated AI approaches, it is possible to identify patients at increased relapse risk in real time, and this initiative provides proactive care. A recent study demonstrated how this works and performs in practice [22-29]. This real-time digital application was successfully integrated into a mental health service to automate service disengagement and non-adherence detection in chronic mental health patients from their MyHR data, Australia’s national health record infrastructure. Integrating such automated monitoring systems into health care would equip health professionals to triage and respond to treatment gaps on a needs basis, *i.e.*, from one size fits all approach to responding when, how and which patient needs support [30-37].

#### 3.2.2 Crowdsourcing and Social Networking Applications

Another source of information of increasing relevance is crowdsourcing applications to harness information and wisdom from groups to address healthcare challenges. In crowdsourcing applications, people provide data or perform tasks, which are aggregated to solve specific problems. A notable example is the PatientsLikeMe community, in which patients provide data about their conditions and treatments, and their aggregated data has been used for health services and pharmaceutical research [38]. To incentivise patients to provide data, social networking processes allow patients to connect, share and learn from each other’s information about treatments, side effects and how their conditions might affect them. Crowdsourcing applications also enable groups of people to receive information and perform tasks to solve problems. For example, cardiopulmonary resuscitation needs to occur in critical time windows, and delays are known to contribute to considerable morbidity and mortality. To address this, a crowdsourced solution involves training a large group of laypeople to administer out-of-hospital CPR and contacting them when needed. When emergency medical services received a call, they sent a text message to proximate laypeople who then provided CPR, resulting in a higher resuscitation rate than control in a randomised controlled trial [35].

#### 3.2.3 Experiences and Outcome Monitoring

Patient-reported experience measures (PREMs) and patient-reported outcome measures (PROMs) are the processes of capturing direct feedback from patients on their symptoms, functioning, health perceptions, and experiences with care received. It is vital to informing care, evaluation and quality improvement. Significant gaps currently exist with the way PREMS and PROMS are operationalised. The standard approach of collecting measures from patients (*i.e.*, instruments, questionnaires, surveys) at fixed time points that rely on patient reports of the past is problematic. Firstly, self-report instruments, even if validated measures, are subject to recall, function overestimation, social desirability effects and state-dependent biases [33]. Secondly, it yields only a periodic “snapshot” rather than a time-varying record of a person’s symptoms, behaviour and functioning that is necessary to detect emerging decline early. The ability to administer these forms of assessment is further diminished in the aftermath of extreme health events, such as the pandemic, as these assessments are typically conducted in person and at treatment facilities that may no longer be feasible due to social distancing requirements or other circumstances.

Digital technologies have the potential to bridge this gap. Using experiential digital applications, we can repeatedly sample experiences, health and pain perceptions of patients through their mobile phones in real-time from the living environment [32] and use it to nudge real-time micro-interventions [28-31].

In addition, the wide availability and advances in wearable digital technologies have made it easier to gather high-quality real-time physiological measurements remotely from patients living environments continuously and over an extended period of time. Such data allows us to examine factors that cause physiological changes within small time spans and also measure the smallest changes that can be achieved by interventions, either behavioural or pharmacological. This combined ability to observe experiences and physiological changes at granular resolution provides opportunities for focused health intervention and research from one size fits all toward personalised approaches [34, 36].

### 3.3 Channels and Mediums

Heart failure patients find attendance and adherence to medical appointments, medications and self-care, which they need to do routinely, and with different domains of the health system, which is burdensome and hard to navigate. Using internet-based digital applications, health systems can now set up alternative channels to reach, organise care and self-help for patients in proactive, accessible, convenient and cheaper ways. Firstly, increasing availability of online appointment booking, electronic referrals, e-scripts applications, and transport apps provide patients with better control and flexible ways to plan and coordinate their logistics of interactions with the health system. Secondly, broader access to telehealth and online therapies provides consults remotely without the need to travel [30]. Lastly, owing to ubiquitous smartphone ownership at a population level and the rapidly reducing digital divide, smartphone apps are becoming programmatic resources for self-help. Since individuals are near their smartphones most of the time, app-based interventions are easy to access on demand. Smartphone apps are ideal for proactively nudging contextual tailored behavioural interventions or medication reminders at the most suitable moments in day-to-day life [28-31]. Mobile phones are not just inexpensive but also a means to reach unreachable populations, thus providing unprecedented opportunities to provide self-care to individuals in everyday life.

Despite the unprecedented opportunities, we need to better understand and resolve barriers to realising the full potential of digital health approaches. At a technical level, approaches used to collect, organise, and present information encounter privacy and quality issues. However, adapting and integrating technology applications into human-centric workflows and processes remain another barrier. Health literacy, self-efficacy, and socio-economic disadvantage, already known mediators of patient engagement with health, can potentially be worsened by digital health applications when they are not carefully co-designed and collaborative with intended users. Lastly, and most importantly, popular commercial digital platforms are increasingly mediating and shaping everyday consumption behaviours and choices of people. The unencumbered application of surveillance and harvesting of personal data in these platforms purely for commercial goals poses the greatest risk to public health, as populations are steered towards behaviours and choices every day, which are potentially detrimental to health, well-being, and autonomy [37].

## CONCLUSION

**Fig. (2):**
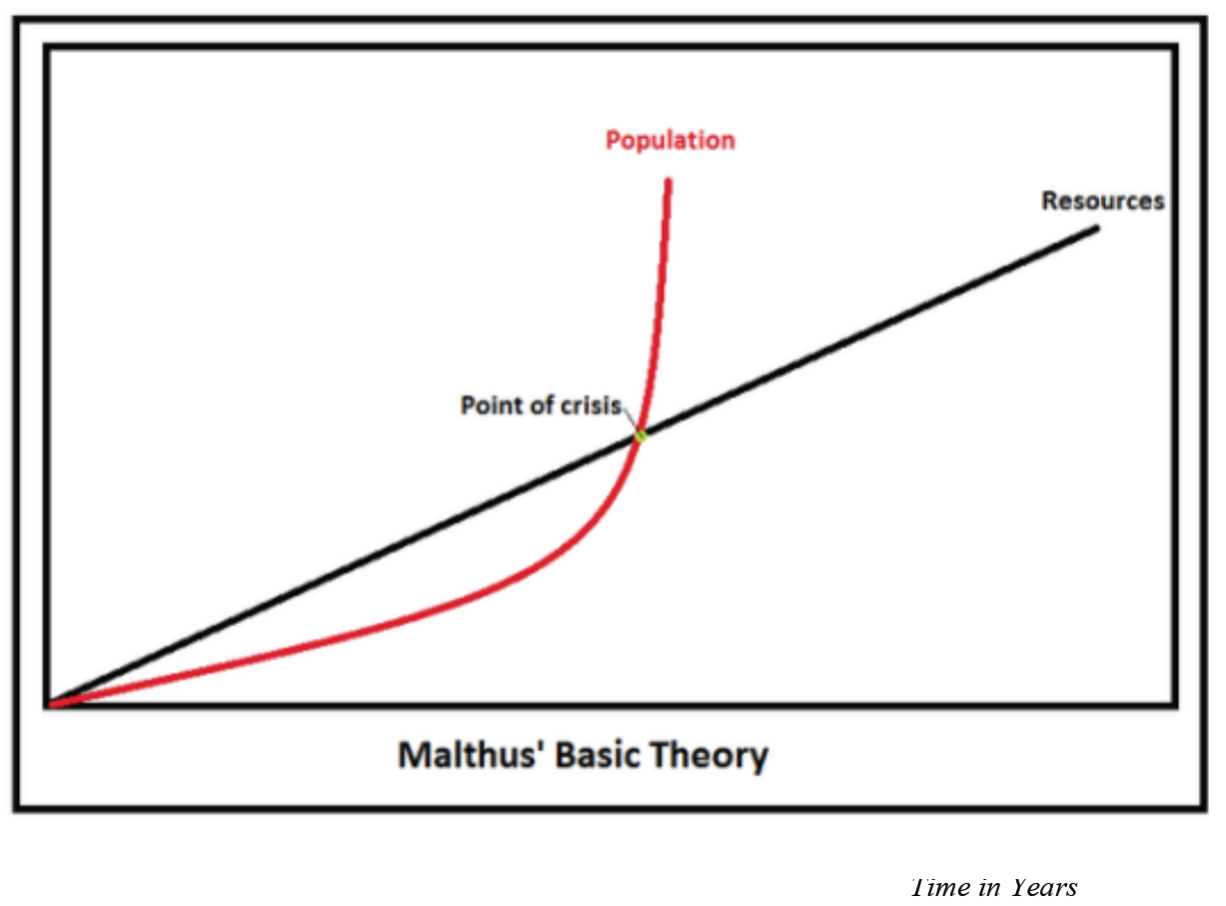
Malthusian Model of Exponential and Proportionality. An exponential rise in congestive heart failure (CHF), with an aging population and migration, is matched by resource growth that rises incrementally. A point is reached when resourcing is unable to match the requirements to service CHF needs. Health technology is a resource that can be designed to match both proportional and exponential terms, the gaps in CHF resourcing. However, there remain gaps in the clinical translation of many technological developments.

Malthusian models are relevant concerning health services due to changes that may be exponential, and the question of proportionality is in question. In a social or non-clinical setting, the variables of population growth and uptake of technologies are more proportional (Fig. **2**). In the medical sphere, they are variable. The authors believe that an important gap lies in health policy links. Product design and commercialisation will encourage investment when the medicolegal framework for handling sensitivities of health data is considered. A framework for possibilities is important to examine free-market entrepreneurship dictating the direction of services as seen with non-medical technology developments. If this occurs, technology can overcome Malthusian concepts and perhaps be ahead of the curve. Imagination innovations must be backed with imaginative safeguards to enhance solutions and reduce patient risk. Furthermore, education campaigns should remove fears and stigma of using health data and encourage uptake (Fig. **2**).

## Figures and Tables

**Table 1:** Ambulatory technologies in heart failure.

Options	Tools	Evidence	Notes	Novel Considerations
**Software** **(Health IT)**	Clinical decision support. Computerized disease registries. Computerized provider order entry. Consumer health IT applications. Electronic medical record systems (EMRs, EHRs, and PHRs). Electronic prescribing. Telehealth.	Clinically utilised (no new evidence needed)	Licensing Cost Maintenance Cross over	Universal integrated and shared health services information.
	Software and Programs.	Variable	Apps Website Social Networking	Health policy advancements.
**Hardware**	Implantable Therapeutic Devices. Implantable Monitoring Devices. Monitoring Devices (*e.g* 24hr Holter).	I A II B IA	PPM, AICD, CRT	Increasing features of implantable therapeutic devices to monitoring.
	Mobile Phone. Computing Devices.	1 A communication Monitoirng and treatment variable	Communication	Home health hubs.

**Abbreviations:** AICD - Automated implantable cardiac defibrillator; CRT – cardiac resynchronisation therapies. Cardiac Permanent Pacemaker.

## Data Availability

Not applicable.

## LIST OF ABBREVIATIONS

CHF: Congestive Heart Failure
HFpEF: Heart Failure with Preserved Ejection Fraction
CCM: Chronic Care Model
PREMs: Patient-Reported Experience Measures
PROMs: Patient-Reported Outcome Measures
